# Lumbar spinal cord ganglioglioma in a 2-year-old Boxer dog

**DOI:** 10.1093/jvimsj/aalag081

**Published:** 2026-05-04

**Authors:** Julia Hauer, Margherita Polidori, Anna Oevermann, Joy Einwaller, Michael Schmohl, Annette Wessmann

**Affiliations:** Neurology Service, Tierklinik Hofheim - IVC Evidensia GmbH, Katharina-Kemmler-Strasse 7, Hofheim am Taunus 65719, Germany; Division of Neurological Sciences, Vetsuisse Faculty, University of Bern, Bern, Switzerland; Division of Neurological Sciences, Vetsuisse Faculty, University of Bern, Bern, Switzerland; Neurology Service, Tierklinik Hofheim - IVC Evidensia GmbH, Katharina-Kemmler-Strasse 7, Hofheim am Taunus 65719, Germany; Neurology Service, Tierklinik Hofheim - IVC Evidensia GmbH, Katharina-Kemmler-Strasse 7, Hofheim am Taunus 65719, Germany; Neurology Service, Tierklinik Hofheim - IVC Evidensia GmbH, Katharina-Kemmler-Strasse 7, Hofheim am Taunus 65719, Germany

**Keywords:** ganglioglioma, glioneuronal tumor, MRI, neuroimaging, spinal cord tumor

## Abstract

A 2-year-old Boxer dog was examined for a 2-week history of progressive, painful right-lateralized paraparesis. Magnetic resonance imaging showed a focal ovoid mass with suspected intramedullary, or less likely, intradural location at the level of L3 vertebral body. Based on the imaging features, neoplasia, and specifically, a nephroblastoma, was suspected. Surgical excision found a difficult to excise mass with indistinct borders. The dog showed marked deterioration postoperatively and was euthanized 3 days later due to suspected myelomalacia. Histopathology and immunohistochemistry of the biopsy revealed that the spinal cord was replaced by a poorly organized proliferation of cells that consisted of both neuronal and glial components, embedded within an edematous neuropil. Immunohistochemistry of the lesion showed positivity for neuronal and astrocytic markers. These findings were consistent with a ganglioglioma, a rare neoplasm in dogs.

## Case description

A 2-year-old intact male Boxer dog was examined because of a 2-week history of mild, progressive right pelvic limb lameness. Neurological examination showed mild paraparesis with marked right-sided lateralization. Paw placement was reduced in the left pelvic limb and nearly absent in the right pelvic limb. Spinal reflex testing showed reduced withdrawal and patellar reflexes in the right pelvic limb. Mild spinal hyperesthesia was elicited on palpation of the mid-lumbar area. The neuroanatomical localization was consistent with a right-sided lesion involving the L4-S1 spinal cord segments and associated nerve roots.

**Figure 1:**
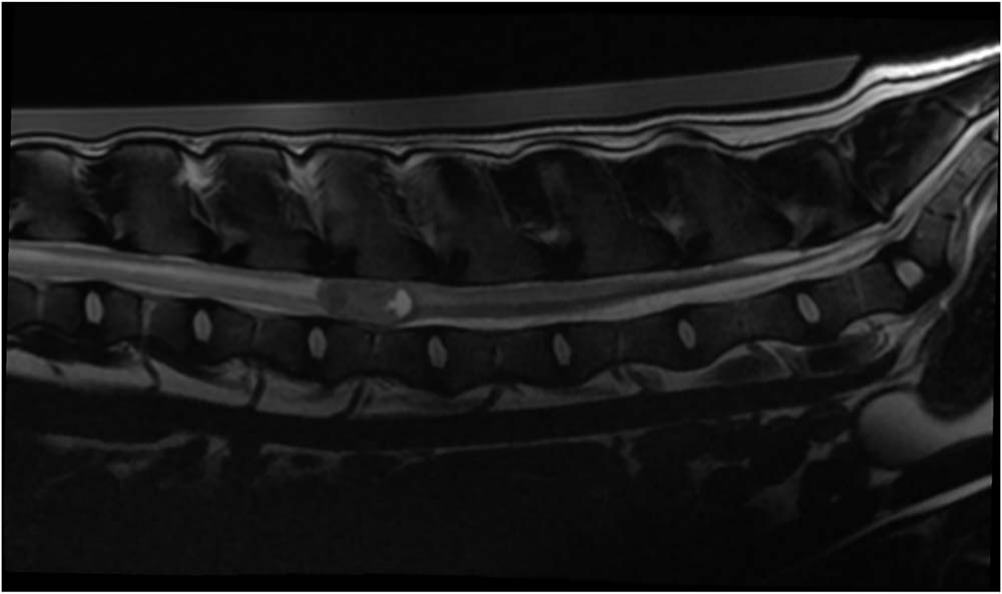
T2W sagittal image and STIR dorsal image showing the T2W iso- to hyperintense lesion with the cyst-like formation in the caudal aspect and the diffuse hyperintensity of the spinal cord parenchyma cranial and caudal to the lesion. Abbreviations: STIR = short τ inversion recovery; T2W = T2 weighted.

**Figure 2:**
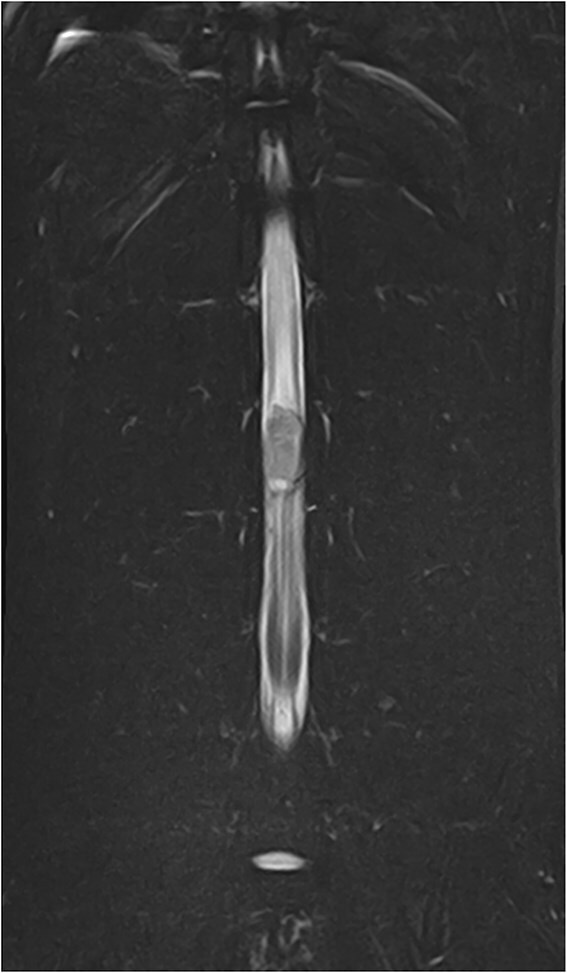
T2W sagittal image and STIR dorsal image showing the T2W iso- to hyperintense lesion with the cyst-like formation in the caudal aspect and the diffuse hyperintensity of the spinal cord parenchyma cranial and caudal to the lesion. Abbreviations: STIR = short τ inversion recovery; T2W = T2 weighted.

**Figure 3:**
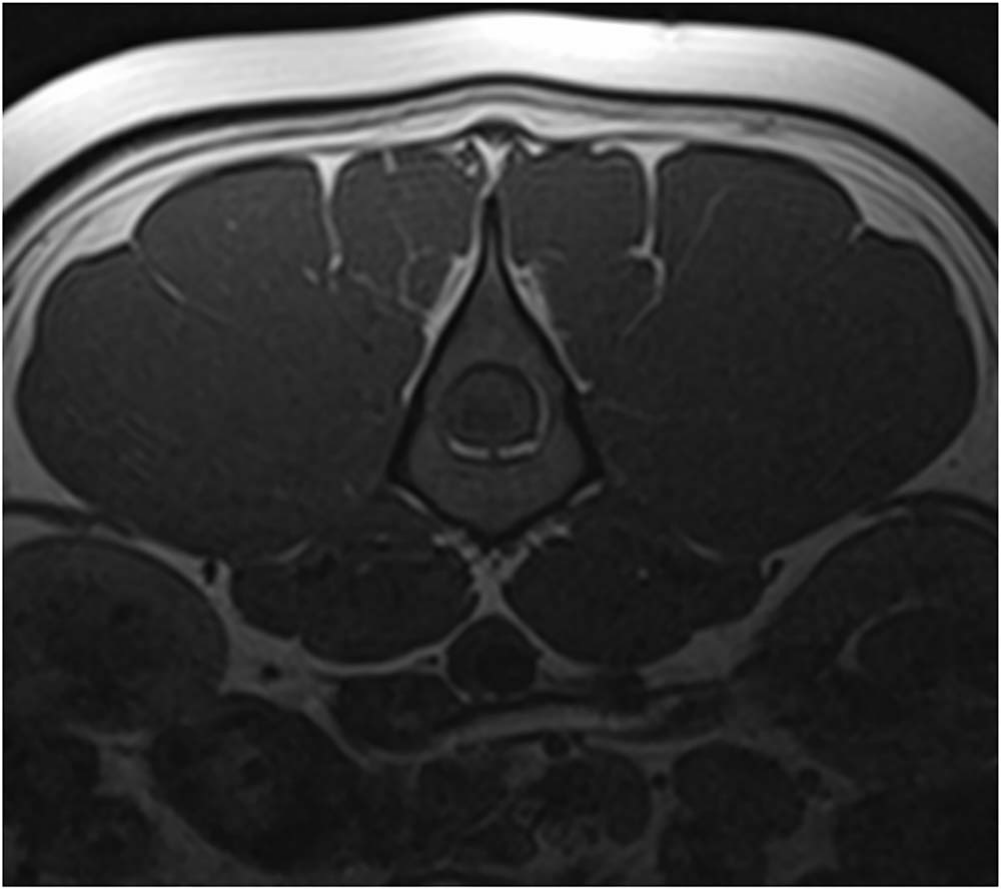
T1W pre- and postcontrast transverse images at the level of L3 showing the extent of the mass lesion with the remaining rim of normal spinal cord tissue and moderate heterogenous contrast enhancement of the mass lesion. Abbreviation: T1W = T1 weighted.

**Figure 4:**
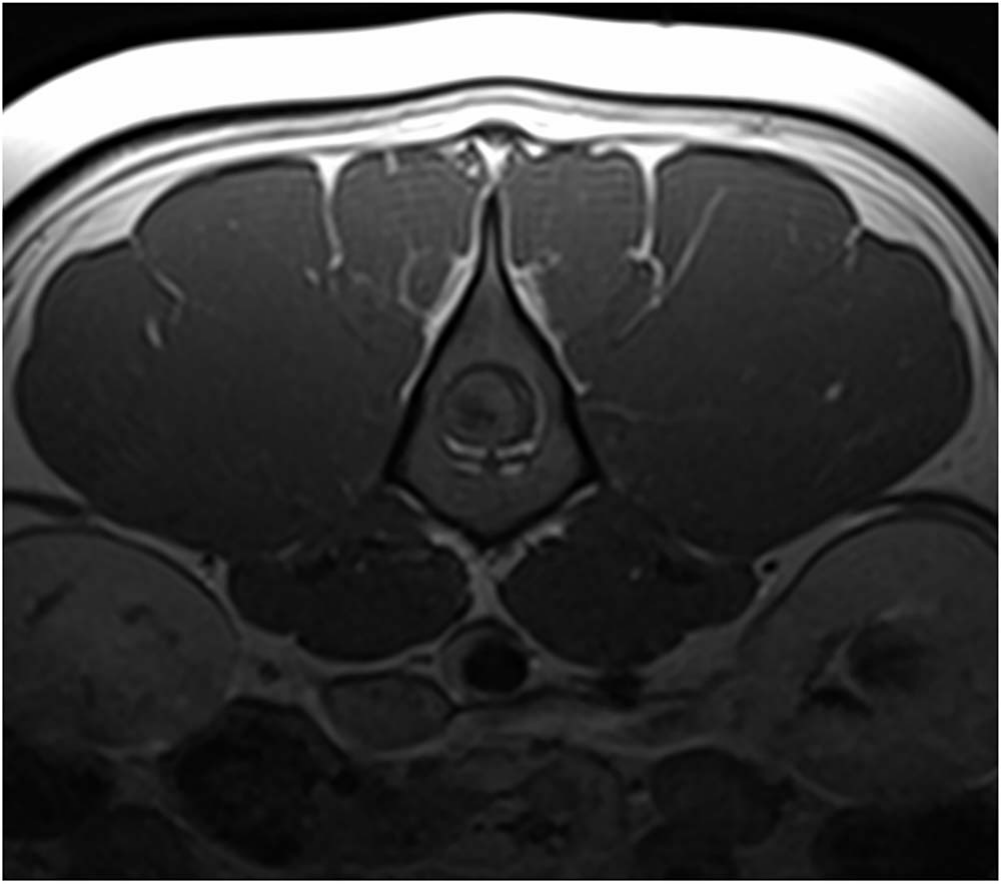
T1W pre- and postcontrast transverse images at the level of L3 showing the extent of the mass lesion with the remaining rim of normal spinal cord tissue and moderate heterogenous contrast enhancement of the mass lesion. Abbreviation: T1W = T1 weighted.

Magnetic resonance imaging (MRI; Canon Vantage Elan, 1.5 Tesla) was carried out under general anesthesia. At the level of the L3 vertebral body, an ovoid, well-demarcated mass, measuring approximately 2.1 × 1.3 cm, occupied nearly the entire cross-sectional diameter of the spinal cord leaving only a thin peripheral rim of residual parenchyma. The mass was iso- to hyperintense on T2-weighted and STIR images, isointense on T1-weighted images, and exhibited moderate heterogeneous enhancement on post-contrast T1-weighted sequences (Figures 1–4). A cyst-like, cerebrospinal fluid (CSF), isointense component was present at the caudal pole of the lesion. No focal dilation of the subarachnoid space (“golf tee sign”) was observed at the cranial and caudal margins. Instead, there was circumferential attenuation of the hyperintense CSF and epidural fat signal on T2-weighted images. The spinal cord cranial, and to a lesser extent caudal to the lesion demonstrated enlargement and diffuse T2-weighted and STIR hyperintensity.

The exact location of the lesion remained inconclusive, as it could represent either a purely intramedullary or an intradural-extramedullary mass with secondary intramedullary invasion.

An additional finding, at the level of L7/S1, was a mild intervertebral disc protrusion and a mild hypertrophy of the ligamentum flavum. The cauda equina nerve roots were mildly compressed, and both intervertebral foramina were slightly narrowed.

Decompressive surgery was elected with the aim of surgical debulking and tissue sampling to achieve a histopathological diagnosis. A right-sided L2-L4 hemilaminectomy followed by a durotomy was performed 7 days after diagnosis. Intraoperatively, the mass was primarily intradural, with apparent extramedullary extension and marked intramedullary infiltration. The mass was excised in small fragments to preserve as much healthy-appearing spinal cord tissue as possible. The abnormal tissue was heterogeneous, consisting of both soft, likely malacic areas, and firmer regions. After resection, consistent with the preoperative MRI observations, the spinal cord appeared severely deformed with a pronounced indentation and only a thin rim of residual spinal cord tissue visible.

Postoperatively, the dog was paraplegic with absent pain perception in the left pelvic limb and preserved pain perception in the right pelvic limb. Three days after surgery, pain perception was absent in both pelvic limbs. Further clinical deterioration occurred, including an ascending cutaneous trunci reflex cut-off, areflexia of both pelvic limbs, inability to maintain sternal recumbency and hyperthermia (39.5*°*C). Descending and ascending myelomalacia was suspected, and the dog was euthanized. The owners declined full postmortem examination, and only the biopsy specimen was submitted for histopathological evaluation. Four biopsy samples from the spinal cord were evaluated, ranging from 0.5 to 0.3 cm in diameter. The tissues were of gray to brown color, and had a soft, almost gelatinous to spinal cord-like consistency. The fragments were fixed in 10% neutral buffered formalin, routinely processed for histology, sectioned at 5 μm, and stained with hematoxylin and eosin.

**Figure 5:**
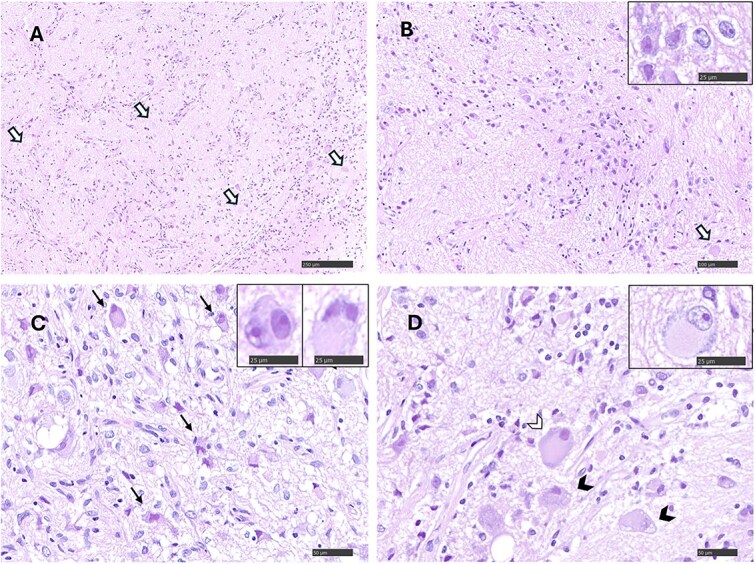
Debulked spinal cord mass tissue, stained with H&E. (A) The normal spinal cord architecture is replaced by moderately densely packed cells lacking an organized architecture and lying in neuropil-like tissue supported by branching vessels. Multiple spheroids are observed (white arrows). Magnification: ×100. (B) The mass is composed of large cells resembling neurons grouped in clusters within a neuropil-like background that contains also glial cells. The white arrow indicates an axonal spheroid. Magnification: ×200. Inset: Neuronal nuclear chromatin is vesicular with prominent nucleoli (magnification ×800). (C) In other areas, dysmorphic neurons (arrows), some of them showing eosinophilic cytoplasm with central chromatolysis, are surrounded by a glial component that is characterized by hypertrophic astrocyte-like cells with epithelioid to fibrillar cytoplasm and pale ovoid nuclei. Magnification: ×400. Inset: Binucleated neurons are occasionally seen. Magnification: ×800. (D) Central chromatolysis (white arrowhead) and intracytoplasmic vacuolization (black arrow heads) of dysmorphic neurons, which are surrounded by glial cells. Magnification: ×400. Inset: Large neuron with central chromatolysis of Nissl substance and small neuron with intranuclear cytoplasmic invagination. Magnification: ×800. Abbreviation: H&E = hematoxylin and eosin.

**Figure 6:**
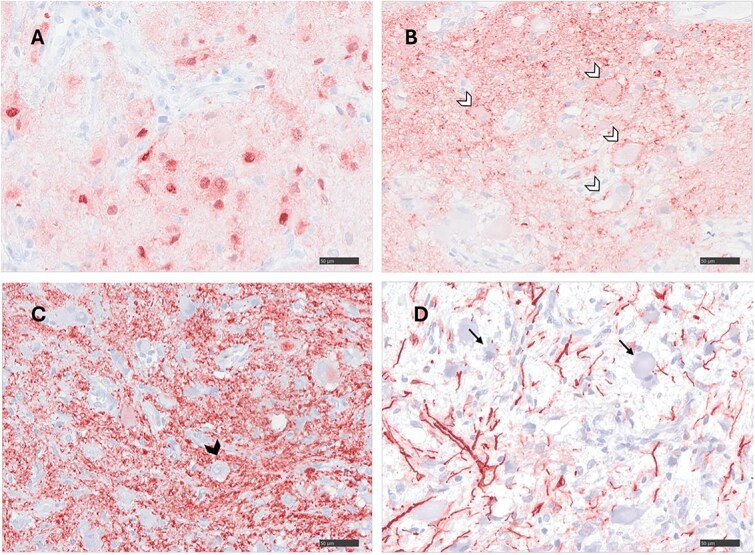
Immunohistochemical stainings of the spinal ganglioglioma. (A) Cluster of tumoral neuronal cells shows intranuclear expression of NeuN (red). (B) Finely granular synaptophysin expression (red) in the neuropil with a perineuronal accentuation indicating synapses (white arrow heads). (C) Strong neurofilament expression in the neuropil indicating presence of axons. Neuronal somas (black arrow head), glial cells and blood vessels remain unstained. (D) Astrocytes near dysmorphic neurons (arrows) express GFAP within their processes (red). Magnification A-D: ×400. Abbreviation: GFAP = glial fibrillary acidic protein

Histologically, no normal spinal cord architecture was visible, but a poorly organized cellular proliferation composed of neuronal and glial elements, embedded in an edematous neuropil was noted in its place (Figure 5A-D). The neuronal component consisted of polygonal cells with abundant eosinophilic cytoplasm, absent to scant Nissl substance, and nuclei containing prominent nucleoli and vesicular chromatin (Figure 5B and C). Scattered immature-appearing cells showed eosinophilic cytoplasm and heterochromatic nuclei (Figure 5C and D). Some neurons appeared dysmorphic and displayed Nissl substance displacement toward the periphery of the cell or vacuolization of the cytoplasm (Figure 5D). Occasional binucleated cells were observed (Figure 5C). Anisocytosis and anisokaryosis were moderate. Mitoses were absent. Degenerative neuronal changes, including swelling, necrosis, chromatin margination, and formation of axonal spheroids, were observed (Figure 5A and B). The glial component comprised hyperplastic astrocyte-like cells with gemnistocytic to fibrillary cytoplasm or bare nuclei (Figure 5C). Mild perivascular inflammatory infiltrates, composed of lymphocytes, plasma cells, and occasional neutrophils, were present within the lesion and in the arachnoid membranes. In one section, the arachnoid membrane appeared hyperplastic with multifocal mineralization. Immunohistochemical labeling confirmed the biphasic neuronal and glial nature of the lesion. The majority of neoplastic nuclei were positive for NeuN (Figure 6A), confirming neuronal differentiation. Neurofilament and synaptophysin immunostaining highlighted numerous axons and synaptic structures, respectively, producing a fine granular signal within the neuropil-like background and surrounding neuronal bodies (Figure 6B and C). Glial fibrillary acidic protein (GFAP) immunostaining showed strong labeling of glial processes, which were multifocally and confluently arranged in a band-like pattern (Figure 6D).

## Discussion

Gangliogliomas are rare, typically benign neoplasms composed of neoplastic glial cells and dysmorphic neurons.^1^ Gangliogliomas might arise from glioneuronal hamartias through neoplastic transformation of the astrocytic component.^2^ Human gangliogliomas are characterized by genetic alterations that activate the MAP kinase pathway.^3^ In humans, these tumors occur within the central nervous system, with a predilection for the temporal lobe and eyes.^1^ They predominantly affect children and young adults,^1^ which aligns with the age of the present case involving a young Boxer dog.

In veterinary medicine, gangliogliomas are exceedingly uncommon with only a few case reports across species, primarily within the central nervous system. They have been described in the spinal cord and the brain of cattle,^4^^,^^5^ the brain of a horse,^6^ and the spinal cord of a hedgehog.^7^ Reports in dogs are rare,^8^^,^^9^^,^^10^^,^^11^^,^^12^ with previously documented cases located in the eyes, thalamus, cerebrum, and the cervical spinal cord.^8^^,^^9^^,^^10^^,^^11^^,^^12^ A single case involving the spinal cord case was mentioned in a larger survey of canine spinal cord neoplasms but without details on the clinical features.^13^

Clinical signs associated with gangliogliomas depend largely on tumor localization. Dogs with forebrain or thalamic involvement presented with seizures,^9^^,^^12^ while a dog with a pituitary ganglioglioma exhibited signs of hyperadrenocorticism.^11^ A 7-year-old German Shepherd with a cervical spinal cord ganglioglioma (C6-C7) showed ambulatory left-sided hemiparesis and proprioceptive deficits in the left thoracic limb and both pelvic limbs.^8^ In the current case, the neurological deficits correlated well with the tumor localization and were also mild, with the dog being ambulatory. The cervical ganglioglioma in the German Shepherd dog was managed conservatively but the dog exhibited rapid and severe clinical deterioration,^8^ comparable to the progression observed in our case over a relatively short period before surgical intervention. This might suggest that once clinical signs of a spinal cord ganglioglioma become evident, neurological deterioration might progress rapidly.

Magnetic resonance imaging features in this case were consistent with a neoplastic process. Considering the dog’s young age, tumor localization, and MRI characteristics, a nephroblastoma was initially considered the most likely differential diagnosis. Nephroblastoma has been reported in both intramedullary and intradural-extramedullary locations with intramedullary extension,^14^ and their imaging features closely resemble those observed here.^15^ However, the MRI signal characteristics also overlapped with descriptions of human gangliogliomas, which are typically hyperintense on T2-weighted images, iso- to hypointense on T1-weighted images, and often present as intramedullary lesions.^16^

Treatment choices in spinal cord tumors are generally palliative, incisional biopsy or cytoreductive surgery, and/or radiotherapy. Often, financial considerations prevent choosing surgical treatment combined with radiotherapy. Cytoreductive surgery has the highest risk for deterioration, yet carries often the best possible prognosis if successful.^14^ Surgical intervention was chosen for this dog to achieve maximal debulking and obtain a histopathological diagnosis to guide further treatment. Intraoperative findings, including the challenging differentiation between dura mater, tumor tissue, and spinal cord parenchyma, were consistent with the preoperative imaging characteristics of the lesion. In human medicine, gross total resection of spinal cord gangliogliomas can often be achieved, and surgical outcomes are generally favorable.^17^ It is our experience that generally, while substantial neurological deterioration can occur postoperatively, dogs recover with persistent neurological deficits.^14^ With hindsight, an incisional biopsy followed by radiotherapy might have been a more reasonable alternative for this dog to avoid such a relevant postoperative deterioration.

Postoperatively, the dog’s neurological status deteriorated rapidly, progressing to paraplegia and loss of pain perception, with clinical suspicion of ascending and descending myelomalacia.

Histologically, the neoplasm displayed the characteristic biphasic pattern of a ganglioglioma, with intermixed neuronal and glial components. The presence of dysmorphic neurons, grade of differentiation, and absence of mitotic activity are consistent with a World Health Organization (WHO) Grade I ganglioglioma.^18^ Similar histological features are reported in cases of ganglioglioma in animals.^8^^,^^12^

This case underscores the importance of considering ganglioglioma as a differential diagnosis in young dogs presenting with neurological deficits involving the spinal cord. Given the difficulty in distinguishing tumor tissue from normal spinal cord parenchyma, surgical treatment should be approached with caution.

## Data Availability

All data are incorporated into the article.

## Off-label antimicrobial declaration

The authors declare no off-label use of antimicrobials.

## Institutional animal care and use committee or other approval declaration

The authors declare no institutional animal care and use committee or other approval was needed.

## Abbreviations

CSF: cerebrospinal fluid
GFAP: glial fibrillary acidic protein
H&E: hematoxylin and eosin
MRI: magnetic resonance imaging
STIR: short τ inversion recovery
T1W: T1 weighted
T2W: T2 weighted
WHO: World Health Organization
